# A Biphasic Glucose Response during an Oral Glucose Tolerance Test Is Associated with Greater Plasma Insulin and GLP-1 Responses and a Reduction in 1-Hour Glucose but Does Not Relate to the Rate of Gastric Emptying in Healthy, Older Adults

**DOI:** 10.3390/nu15183889

**Published:** 2023-09-06

**Authors:** Ryan J. Jalleh, Chinmay S. Marathe, Laurence G. Trahair, Karen L Jones, Michael Horowitz

**Affiliations:** 1 Centre of Research Excellence in Translating Nutritional Science to Good Health, Adelaide Medical School, The University of Adelaide, Adelaide, SA 5000, Australia; 2 Endocrine and Metabolic Unit, Royal Adelaide Hospital, Adelaide, SA 5000, Australia; 3 Endocrine and Diabetes Services, Northern Adelaide Local Health Network, Adelaide, SA 5112, Australia

**Keywords:** gastric emptying, glucose tolerance, glycaemia, insulin sensitivity, oral disposition index, type 2 diabetes

## Abstract

Background: The pattern of the plasma glucose response curve during an oral glucose tolerance test (OGTT) is of prognostic significance with “biphasic” when compared with “monophasic” patterns being associated with greater insulin sensitivity/secretion and a reduced risk of progression to diabetes. The relationships of the glucose response curves with gastric emptying and incretin hormone secretion are not known. Methods: Thirty-six adults (age > 65 years) without known diabetes consumed a 300 mL drink containing 75 g glucose and 150 mg C^13^-acetate at baseline and follow-up after 5.8 ± 0.1 years. Plasma glucose, glucagon-like peptide-1 (GLP-1), glucose independent insulinotropic polypeptide (GIP) and insulin were measured, and participants classified according to the pattern of their glucose response. Gastric emptying was measured on breath samples (stable isotope breath test). Results: At baseline, 22 participants had a “monophasic” and 14 a “biphasic” glucose response. The 1 h plasma glucose response curve was greater and the GLP-1 AUC_0–120 min_ and insulin secretion lower in the monophasic group. There were no differences in gastric emptying, GIP or insulin sensitivity. At the follow-up, the 1 h glucose response curve was greater again, while GLP-1 AUC_0–120 min_ was lower in the monophasic group. Conclusions: A biphasic curve is associated with a higher 60 min glucose response curve and increases in GLP-1, but no difference in either GIP or gastric emptying.

## 1. Introduction

It is not widely appreciated that, in addition to the diagnostic implications of baseline, 60 min and 120 min plasma glucose levels [1], the shape of the glucose response curve during an oral glucose tolerance test (OGTT) provides useful insights into insulin secretion and sensitivity, even in individuals with normal glucose tolerance [2,3,4,5,6]. The shape has been classified according to whether it exhibits either (i) an incessant rise in glucose, (ii) a monophasic curve, (iii) a biphasic curve or (iv) a more complex curve [4]. The incessant/progressive rise in glucose appears to be associated with the greatest risk of dysglycaemia and incident diabetes [4]. A monophasic curve, as evaluated in Latino youths [3], youths with obesity [4] and autoantibody-positive relatives of people with type 1 diabetes [5], is associated with impaired glucose tolerance, reduced insulin sensitivity and secretion and an increased risk of future diabetes when compared with biphasic, or more complex, curves. In contrast, increasing the complexity of the shape (i.e., greater numbers of glucose peaks) is associated with better glucose tolerance and beta cell function [6]. While these associations are well established, the mechanisms accounting for these differences in glucose patterns and their implications for glucose tolerance remain poorly defined [2]. Specifically, there is no information about the potential roles of the rate of gastric emptying (GE) and the incretin hormones.

GE, for which there is a substantial inter- (~1–4 kcal/min) but lesser intra-individual variation, is now recognised to be a major determinant of postprandial glucose, accounting for ~35% of the post-prandial blood glucose response [7]. In some racial groups predisposed to the development of type 2 diabetes (T2D), gastric emptying is accelerated [8,9]. Moreover, uncomplicated T2D, in contrast to longstanding, complicated T2D, is associated with more rapid, rather than delayed, GE [10].

Glucagon-like peptide-1 (GLP-1) and glucose-independent insulinotropic polypeptide (GIP), released from the small intestine, increase insulin sensitivity and secretion and, in the case of GLP-1, suppress glucagon [11]. An increase in small intestinal glucose delivery, as when gastric emptying is more rapid, is associated with greater GIP and GLP-1 secretion. Studies in which glucose has been delivered directly into the small intestine indicate that the patterns of response differ [12]. The rate of small intestinal glucose delivery correlates linearly with a rise in GIP, whereas the GLP-1 response is minimal at lower rates of glucose delivery (1–2 kcal/min), but substantial at rates of 3–4 kcal/min [12]. In older adults without diabetes, we have shown that glucose-stimulated GLP-1 and GIP concentrations correlate, even after a period of ~6 years, but ‘healthy’ aging is associated with modest reductions in fasting GLP-1 and GIP, as well as glucose-stimulated GLP-1 [13]. In addition to its glucose-dependent insulinotropic and glucagonostatic properties, GLP-1 also plays a physiological role in slowing GE [14] and the rate of GE in both healthy and T2D individuals appears to be determined in part by the GLP-1 response to intestinal nutrients. GIP, in contrast, has no effect on GE [15].

We performed, in older individuals (>65 years) without known diabetes, a cross-sectional, longitudinal evaluation of the association of the shape of the glucose response curve with GLP-1 and GIP secretion, and GE as well as insulin secretion and sensitivity. We hypothesised that a relatively more rapid rate of GE would be associated with a monophasic rather than a biphasic curve, reflecting the relatively faster influx of glucose into the small intestine. We also hypothesised that plasma GLP-1 and GIP concentrations would be greater in the monophasic group in this cohort as a compensatory response to the glycaemic excursion.

## 2. Materials and Methods

### 2.1. Participants

Information relating to the relationship of blood pressure to the rate of gastric emptying of a glucose drink in this cohort of older individuals has been previously reported [16]. At the time of the initial study, participants were 65–90 years old, without a history of diabetes. No participant was taking medication known to influence gastric emptying, and smoking (which may slow GE [17]) was prohibited on the morning of the studies. Individuals with a history of significant cardiac, respiratory, gastrointestinal, renal or hepatic disease, previous gastrointestinal surgery (apart from appendicectomy or cholecystectomy) or with an alcohol consumption of >20 g per day, were excluded. 

Following the initial study, participants were invited to attend a follow-up study after a mean interval of 5.8 ± 0.1 (SEM) years.

All participants gave informed consent for their participation. The study was conducted in accordance with the Declaration of Helsinki, and the protocol was approved by the Human Research Ethics Committee of the Royal Adelaide Hospital.

### 2.2. Protocol

The protocol at the initial and follow-up studies was identical. Participants presented at 8.30 am after an overnight fast (14 h for solids and 12 h for liquids) when an intravenous cannula was inserted into the antecubital vein to facilitate blood sampling. A drink containing 75 g of glucose and 150 mg of 13C-acetate (Cambridge Isotope Laboratories, Tewksbury, MA, USA), made up to 300 mL with water at room temperature was then consumed within 5 min—t = 0 was defined as the time of completion of the drink. 

### 2.3. Biochemical Measurements

Plasma glucose (hexokinase method), insulin (RRID: AB_2877672, ELISA, Diagnostics 10–1113, Mercodia, Uppsala, Sweden), GLP-1 (RRID: AB_2757816, GLPIT-36HK, Millipore, Billerica, MA, USA) and GIP (RRID: AB_518352, In-house assay, Peninsula Laboratories, CA, USA, cat. no T-4052 rabbit anti-GIP [human] antiserum) were measured at baseline and t = 15, 30, 45, 60, 90 and 120 min.

### 2.4. Insulin Secretion, Sensitivity and Oral Disposition Index

Insulin secretion was estimated using the “insulinogenic index” of Δinsulin0–30/Δglucose0–30, insulin sensitivity using 1/fasting insulin and the oral disposition index (oDI) calculated using the product of insulin secretion and insulin sensitivity, i.e., 1/fasting insulin × Δinsulin0–30/Δglucose0–30 [18].

### 2.5. Gastric Emptying

Exhaled breath samples were collected before ingestion of the drink (t = −3 min) and then every 5 min for the first hour (commencing at t = 5 min) followed by every 15 min for the next 3 h. The 13CO2 concentration in the breath samples was measured using an isotope ratio mass spectrometer (ABCA 20/20; Europa Scientific, Crewe, UK), and the gastric 50% emptying time (T50) was calculated [19]. Wagner–Nelson analysis was utilised to generate a gastric emptying curve from the percentage of 13CO2 measured in breath samples and the gastric emptying rate (kcal/min) was calculated [19].

### 2.6. Statistical Analysis

**Figure 1:**
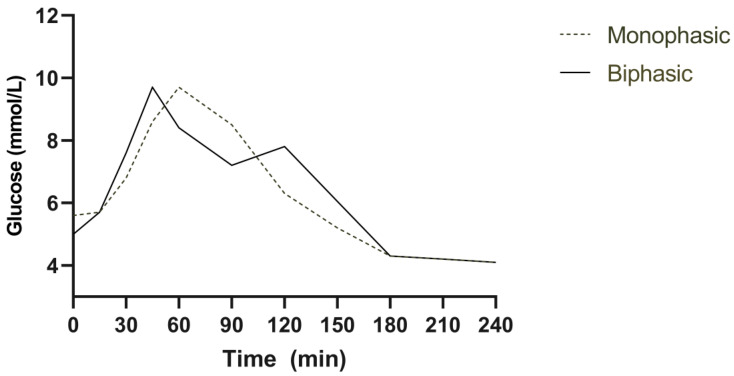
Monophasic (dotted line) and biphasic (solid line) glucose response curves following a 75 g oral glucose tolerance test.

Glucose tolerance was classified according to The Expert Committee on Diagnosis and Classification of Diabetes Mellitus definitions [20]. Impaired fasting glucose was defined as fasting plasma glucose 5.6–6.9 mmol/L, impaired glucose tolerance as a 2 h value post-OGTT of 7.8–11.0 mmol/L and type 2 diabetes (T2D) as fasting glucose ≥ 7.0 mmol/L or 2 h plasma glucose of ≥11.1 mmol/L post-OGTT. Total areas under the curve (AUCs) between t = 0 and 120 min were calculated using the trapezoidal rule. Participants were subdivided into groups according to their plasma glucose response—either incessantly rising glucose, a monophasic response (a gradual increase in plasma glucose to a peak followed by a subsequent decline), a biphasic response (a gradual increase in plasma glucose to a peak, followed by a fall of ≥0.25 mmol/L, and then a second rise of ≥0.25 mmol/L within 2 h) [4] or a more complex pattern (Figure 1) [6]. If a better fit of normal distribution was obtained through log transformation (log 10), this was performed before the statistical analysis. Normality was confirmed with a Shapiro–Wilk test. Differences were analysed using an unpaired Student’s *t*-test and shown as means ± SEM. A *p*-value of less than 0.05 was considered a significant difference.

## 3. Results

### 3.1. Baseline Measurements

Participant characteristics are summarised in the table below [Table 1]. The monophasic group were older in age but both groups had similar height, weight and body mass index.

### 3.2. Plasma Glucose

Forty-one participants (17 women, 24 men) were recruited: 19 (46%) had normal fasting glucose and normal glucose tolerance, 2 (5%) had impaired fasting glucose, 12 (29%) had impaired glucose tolerance, 3 (7%) had both impaired fasting glucose and impaired glucose tolerance and 5 (12%) had undiagnosed type 2 diabetes—in these 5 participants, their general practitioners were notified of the results and they were excluded from the analysis. Of the remaining 36 individuals (who all attended the following visit), 22 participants (61%) had a “monophasic” and 14 (39%) a “biphasic” glucose response. None had either an incessantly rising pattern or a more complex pattern. The monophasic group was modestly older (*p* < 0.001). While fasting, glucose was not different in both groups (*p* = 0.67); the 1 h post-OGTT plasma glucose response was greater in the monophasic vs. biphasic group 9.5 ± 0.5 mmol/L vs. 8.0 ± 0.5 mmol/L (*p* = 0.04). 

### 3.3. Insulin Secretion and Sensitivity

In the monophasic, there was an approximate two-fold reduction in insulin secretion compared to the biphasic (10.4 ± 1.1 vs. 20.9 ± 4.3, *p* = 0.03) group. There were no differences in either insulin sensitivity or the oral disposition index between the two groups [Table 2].

### 3.4. Plasma GLP-1 and GIP

In the monophasic group, both peak GLP-1 (*p* = 0.007) and the GLP-1 AUC_0–120 min_ OGTT (*p* = 0.02) were lower. In contrast, there were no differences in plasma GIP between the two groups.

### 3.5. Gastric Emptying

Gastric emptying was comparable in the two groups, whether expressed as a caloric rate (monophasic 1.15 ± 0.04 vs. biphasic 1.10 ± 0.05 kcal/min, *p* = 0.45) or as the gastric 50% emptying time (T50) (135 ± 6 min vs. 141 ± 8 min, *p* = 0.52).

### 3.6. Follow-Up

At the follow-up, 6 (17%) individuals with an initial monophasic response had a biphasic response and 8 (22%) individuals with a biphasic response had a monophasic response; the remaining 22 (61%) had the same response curve. 

There was a modest reduction in fasting glucose (0.2 ± 0.1 mmol/L, *p* = 0.036) at the follow-up visit, but no difference in 1 h/2 h plasma glucose responses, glucose peaks or AUCs. As with the initial visit, a monophasic glucose pattern was associated with a higher 1 h plasma glucose response (*p* = 0.01) but not 2 h (*p* = 0.78) or fasting glucose (*p* = 0.09). There was, again, no difference in GIP, while GLP-1 was lower in the monophasic compared with the biphasic group. There were no differences in insulin secretion (*p* = 0.20), sensitivity (*p* = 0.44) and the oral disposition index (*p* = 0.15) [Table 3].

Gastric emptying, again, was not significantly different in the monophasic vs. biphasic group. The gastric 50% emptying time was 104 ± 10 min vs. 131 ± 17 min (*p* = 0.16) and the caloric rate was 1.78 ± 0.16 vs. 1.36 ± 0.16 kcal/min (*p* = 0.11). 

## 4. Discussion

In this cross-sectional and longitudinal study, a biphasic glucose response to an oral glucose tolerance test was associated with a reduction in the 60 min glucose response and increases in insulin secretion and plasma GLP-1, but not differences in insulin sensitivity, plasma GIP or gastric emptying at both baseline and follow-up when compared with a monophasic response. These observations suggest that an increased GLP-1 response may be central to the reduced risk of dysglycaemia known to be associated with biphasic, compared to monophasic, glucose responses [2,4]. The absence of an incessantly rising pattern in our cohort is not surprising given that this pattern is associated with the most severe impairments in glucose metabolism where individuals would have been likely to be excluded from our study due to a diagnosis of T2D. The monophasic group were older than the biphasic group but this could be explained by older age being associated with reduced insulin secretion [21] and, therefore, monophasic responses.

The demonstrated association of a biphasic glucose curve with a greater GLP-1 response is novel and also provides an explanation for the observed increase in insulin secretion and reduction in 1 h glucose responses. We speculate that the greater GLP-1 response in individuals with biphasic curves reflects an intrinsic increase in GLP-1 secretion, particularly as there was no difference in GE [22]. In a prior study [23] in youths with obesity, neither GIP or GLP-1 responses differed significantly in biphasic or monophasic cohorts; however, the GLP-1 response to oral glucose is known to be reduced in individuals with obesity, which may account for this difference in observations [24]. We suggest that the elevated GLP-1 response accounts for the observed increases in insulin secretion and reduction in blood glucose during the OGTT in healthy, older individuals with biphasic curves.

The fact that the pattern of the glucose response curve changed in 39% of individuals after 5 years of follow-up is consistent with a prior longitudinal study [2], in which follow-up was for 3 years. While the mechanisms underlying the change in this glucose response curve are unknown, our study suggests that it is most unlikely to be related to either gastric emptying or incretin hormone secretion. Factors that warrant evaluation include changes in the gut microbiota that have been associated with altered glucose homeostasis [25]. In addition, it should be appreciated that the oral glucose tolerance test is associated with some intra-individual variability, with 1 h blood glucose typically varying by 1.9 mmol/L, which may have led to an altered glucose response curve [26].

The observation that the biphasic curve is associated with a reduction in the 1 h glucose response is of potential importance given that an elevated 1 h glucose post-OGTT (≥8.6 mmol/L) is now recognised as a strong predictor of future type 2 diabetes, outperforming both the 2 h glucose response and HbA1c [27]. In the monophasic group, the mean 1 h glucose response was ≥8.6 mmol/L at both baseline and follow-up, whereas it was <8.6 mmol/L in the biphasic group. It would, accordingly, be of interest to know how the shape of the glucose curve compares with an elevated 1 h glucose response as a predictor of future type 2 diabetes and whether, in individuals with an elevated 1 h glucose response, GLP-1 secretion is reduced.

Our study is the first to evaluate the shape of the glucose curve longitudinally, concurrently with measurements of incretin hormones and GE. Furthermore, Wagner–Nelson analysis was used to evaluate GE as this method enables isotope breath tests to be more comparable to the “gold-standard” of scintigraphy [19].

## 5. Limitations

We had a modest sample size and used surrogate markers of insulin secretion and sensitivity rather than the hyperinsulinaemic, euglycaemic clamp study [28]. We also did not measure the OGTT repeatedly or measure glucagon, glucose absorption or the changes in gut microbiota.

## 6. Conclusions

In summary, a biphasic plasma glucose curve following a 75 g oral glucose tolerance test is associated with a reduction in the 60 min glucose response and increases in GLP-1, but no difference in GIP or gastric emptying when compared to a monophasic response in older individuals without diabetes.

## Figures and Tables

**Table 1:** Participant characteristics at baseline analysed using an unpaired Student’s *t*-test. Data are mean ± SEM.

	Monophasic	Biphasic	*p*-Value
Age (years)	77.3 ± 0.7	69.2 ± 0.9	<0.001 *
Height (m)	1.68 ± 0.02	1.67 ± 0.03	0.94
Weight (kg)	72.0 ± 2.9	73.4 ± 3.4	0.77
Body mass index (kg/m^2^)	25.6 ± 0.7	26.0 ± 0.6	0.62

* statistically significant difference.

**Table 2:** Participant glucose, insulin and incretin concentrations at the initial study analysed using an unpaired Student’s *t*-test. Data are mean ± SEM.

		Monophasic (n = 22)	Biphasic (n = 14)	*p* Value
Glucose (mmol/L)	Fasting	5.8 ± 0.9	5.7 ± 1.0	0.67
	1 h	9.5 ± 0.5	8.0 ± 0.5	0.04 *
	2 h	7.5 ± 0.4	7.3 ± 0.4	0.66
	Peak	10.6 ± 0.4	9.6 ± 0.3	0.01 *
	AUC_0–60_	522 ± 15	472 ± 16	0.04 *
	AUC_0–120_	1032 ± 43	920 ± 37	0.08
Insulin (pmol/L)	Fasting	3.8 ± 0.4	4.9 ± 0.6	0.13
	1 h	54.5 ± 5.4	67.2 ± 13.7	0.40
	2 h	44.9 ± 5.5	54.1 ± 9.1	0.36
	Peak	63.2 ± 7.0	75.7 ± 13.3	0.37
	AUC_0–60_	1942 ± 211	2712 ± 488	0.17
	AUC_0–120_	3568 ± 352	4522 ± 859	0.32
	Insulin secretion	10.4 ± 1.1	20.9 ± 4.3	0.03 *
	Insulin sensitivity	0.32 ± 0.04	0.24 ± 0.02	0.14
	Oral disposition index	3.2 ± 0.4	4.5 ± 0.8	0.13
GLP-1 (pmol/L)	Fasting	20.8 ± 1.2	21.3 ± 1.5	0.82
	1 h	30.7 ± 1.8	33.3 ± 2.4	0.37
	2 h	24.3 ± 1.4	30.3 ± 2.3	0.02 *
	Peak	36.2 ± 2.1	49.4 ± 4.8	0.007 *
	AUC_0–60_	1846 ± 97	2290 ± 171	0.02 *
	AUC_0–120_	3421 ± 165	4190 ± 288	0.02 *
GIP (pmol/L)	Fasting	20.0 ± 1.7	19.5 ± 1.8	0.83
	1 h	52.1 ± 3.8	49.2 ± 3.7	0.60
	2 h	52.7 ± 3.9	53.8 ± 3.5	0.84
	Peak	56.4 ± 4.1	55.7 ± 3.7	0.91
	AUC_0–60_	2668 ± 189	2436 ± 148	0.39
	AUC_0–120_	5863 ± 424	5546 ± 354	0.60

* statistically significant difference.

**Table 3:** Participant glucose, insulin and incretin concentrations at the follow-up study analysed using an unpaired Student’s *t*-test. Data are mean ± SEM.

		Monophasic (n = 24)	Biphasic (n = 12)	*p* Value
Glucose (mmol/L)	Fasting conc.	5.6 ± 0.1	5.4 ± 0.2	0.09
	1 h	10.0 ± 0.4	7.9 ± 0.3	0.01 *
	2 h	7.4 ± 0.4	7.6 ± 0.4	0.78
	Peak	10.6 ± 0.3	9.2 ± 0.4	0.02 *
	AUC_0–60_	498 ± 13	457 ± 14	0.06
	AUC_0–120_	1027 ± 32	936 ± 39	0.10
Insulin (pmol/L)	Fasting	5.2 ± 0.5	4.9 ± 0.8	0.76
	1 h	76.4 ± 10.2	50.6 ± 11.2	0.11
	2 h	72.0 ± 12.3	52.1 ± 8.5	0.19
	Peak	101.4 ± 14.9	80.3 ± 13.3	0.42
	AUC_0–60_	2500 ± 300	2676 ± 587	0.79
	AUC_0–120_	7472 ± 1044	5920 ± 923	0.27
	Insulin secretion	12.4 ± 1.6	17.4 ± 3.4	0.20
	Insulin sensitivity	0.23 ± 0.02	0.28 ± 0.07	0.44
	Oral disposition index	2.7 ± 0.4	3.9 ± 0.7	0.15
GLP-1 (pmol/L)	Fasting	14.6 ± 0.9	16.8 ± 1.2	0.16
	1 h	25.5 ± 1.4	31.7 ± 3.1	0.04 *
	2 h	20.2 ± 1.3	25.2 ± 2.3	0.06
	Peak	30.9 ± 1.8	44.4 ± 4.7	0.003 *
	AUC_0–60_	1422 ± 73	2036 ± 188	0.001 *
	AUC_0–120_	2759 ± 116	3837 ± 335	0.001 *
GIP (pmol/L)	Fasting	16.4 ± 1.2	18.3 ± 1.5	0.36
	1 h	49.1 ± 2.6	49.3 ± 4.3	0.97
	2 h	49.0 ± 3.0	53.6 ± 3.7	0.36
	Peak	53.0 ± 3.0	56.7 ± 4.0	0.47
	AUC_0–60_	2384 ± 127	2534 ± 189	0.51
	AUC_0–120_	5365 ± 298	5662 ± 426	0.57

* statistically significant difference.

## Data Availability

The data presented in this study are available on request from the corresponding author. The data are not publicly available due to participant confidentiality.
